# The role of general self-efficacy and generalized anxiety symptoms in the relationship between exercise adherence and psychological resilience among college students

**DOI:** 10.3389/fpsyg.2026.1809819

**Published:** 2026-07-08

**Authors:** Qi Liu, Peijun Wei, Chaofan Chen, Weidong Zhu, Hailong Li, Bo Li

**Affiliations:** 1 Institute of Sports Science, Nantong University, Nantong, China; 2 Qilu Institute of Technology, Jinan, China; 3 Division of P.E., Tsinghua University, Beijing, China; 4 IDG/MCGOVERN Institute for Brain Research, Tsinghua University, Beijing, China; 5 School of Physical Education and Sports, Soochow University, Soochow, China; 6 Department of Physical Education, Shenzhen Polytechnic University, Shenzhen, China

**Keywords:** exercise adherence, general self-efficacy, generalized anxiety symptoms, psychological resilience, university students

## Abstract

**Objective:**

This study aimed to examine the associations between exercise adherence and psychological resilience among university students and to explore the chain mediating roles of general self-efficacy and generalized anxiety symptoms within these associations.

**Methods:**

A total of 24,979 university students were recruited using a stratified, cluster, and multi-stage sampling method. Data on exercise adherence, general self-efficacy, generalized anxiety symptoms, and psychological resilience were collected via an online questionnaire platform Wenjuanxing.

**Results:**

Exercise adherence was significantly and positively associated with psychological resilience, with a total effect of 0.670, a direct effect of 0.382, and an indirect effect of 0.288. Exercise adherence was significantly and positively correlated with general self-efficacy and significantly and negatively correlated with generalized anxiety symptoms. General self-efficacy was significantly and positively correlated with psychological resilience), and significantly and negatively correlated with generalized anxiety symptoms. Generalized anxiety symptoms were significantly and negatively correlated with psychological resilience, and general self-efficacy mediated the association between exercise adherence and psychological resilience, generalized anxiety symptoms likewise mediated this relationship. A mediation effect of general self-efficacy and generalized anxiety symptoms was identified between exercise adherence and psychological resilience.

**Conclusion:**

Exercise adherence is directly and positively associated with psychological resilience in university students, and it shows an indirect association with resilience through higher general self-efficacy, which in turn is associated with lower generalized anxiety symptoms.

## Introduction

Psychological resilience (PR) refers to an individual’s capacity to rapidly recover from and adapt to adversities such as stress, setbacks, and trauma (Davidov et al., 2015; Hu and Gan, 2008). As a crucial psychological trait, resilience is positively correlated with indicators of mental health and plays a significant role in mitigating psychological burdens and enhancing an individual’s mental well-being (Su et al., 2023). Mental health issues among university students in China have become increasingly prominent, with anxiety ranking foremost among various mental health concerns (Fu and Zhang, 2021). University students face a wide array of stressors. Intensive academic workloads, an increasingly competitive job market, and a rapidly changing social environment collectively pose threats to their mental health. Research indicates that individuals with high resilience are better able to manage negative emotions, maintain a positive emotional state and outlook on life, and consequently improve their quality of life (Shi et al., 2015). Therefore, well-developed resilience is thought to be associated with better emotional regulation and environmental adaptability in university students, thereby safeguarding their mental health (Liu et al., 2025b). Individuals with low resilience often exhibit inadequate coping skills in the face of adversity, are less likely to employ positive coping strategies, and are more susceptible to symptoms such as anxiety (Li and Xie, 2022). In recent years, PR among university students in China has declined (Shi et al., 2023). Consequently, investigating the factors that relate to the PR of university students holds significant practical importance.

Exercise adherence (EA) refers to the sustained, regular physical activity behavior that individuals maintain by investing both emotional and volitional effort (Wang et al., 2016). With the accelerating pace of modern life, a growing number of people are neglecting the importance of physical exercise and finding it challenging to cultivate the habit of long-term adherence to exercise (Chen et al., 2021). Studies have shown that a chronic lack of exercise often leads to increased fatigue during daily activities, diminished mental state, and consequently a heightened risk of developing psychological problems (Nelson et al., 2012; Lavie et al., 2019; Stea et al., 2022). Conversely, EA has been associated with cognitive and attentional abilities, fosters greater tolerance and perseverance in difficult situations, and enables a more composed response to adverse events (Zhang et al., 2022). Previous research indicates that active, regular physical exercise is significantly associated with the psychological well-being of university students (Ye et al., 2023). As a distinct social group, university students’ physical and mental health has garnered widespread attention (Ni et al., 2025). University students’ leisure time is often spent on electronic devices, reducing time for physical activity and lowering the likelihood of maintaining long-term, regular exercise (Liu et al., 2025a). Academic workload and examination pressures further hinder university students’ ability to maintain regular physical activity, while a lack of interest in exercise also affects their adherence to it (Jin et al., 2022; Yu, 2025; Li et al., 2025). Regular physical exercise may show a strong association with PR; however, research on the mechanisms underlying the association between EA and resilience remains scarce (Zhang et al., 2025; White et al., 2024; Li and Huang, 2025). Therefore, from an EA perspective, this study examines the factors influencing PR among university students.

## Literature review and research hypotheses

### Relationship between exercise adherence and psychological resilience

A single instance of physical exercise yields limited effects; sustained, regular EA contributes to improvements in an individual’s physical and mental health. EA is not merely about the behavior itself but also involves complex psychological processes. It can lead to improvements not only in physical health but also in the individual’s psychological well-being (Tian, 2024). Studies have found a potentially close relationship between physical exercise and PR. Adherence to regular physical exercise may enhance brain function, regulate behavior, and thereby be positively related to PR (Li and Liang, 2025). In this process, EA is considered a crucial behavioral input, and its level may be associate with changes in PR. Individuals with low PR often struggle to cope with difficulties positively and consequently find it hard to meet the demands of exercise tasks. In contrast, individuals with high PR possess more stable self-regulatory abilities and are more willing to use positive coping strategies to overcome difficulties (Jin and Dong, 2023). Although existing academic research on PR provides a theoretical foundation for this study, research specifically on EA among university students’ PR remains in its early stages and warrants further exploration (Tang and Wang, 2024). The resilience framework model posits that PR is not a fixed, static trait but rather a dynamic developmental process. It involves continuous interaction between the individual and adversity, through which active self-reorganization ultimately leads to positive adaptation (Kumpfer, 1999). Based on this, the relationship of EA on PR can be understood as a dynamic, self-reinforcing psychological process. This process relies on the individual’s volitional and emotional investment, strengthens psychological adaptability, and continuously affects their PR. Based on the above analysis, the following hypothesis is proposed:

*Hypothesis H1*: Exercise adherence has a significant positive statistical association with psychological resilience.

### The mediating role of general self-efficacy between exercise adherence and psychological resilience

General self-efficacy (GSE) refers to an individual’s confidence in their ability to use acquired skills to complete tasks or perform behaviors in specific situations (Zhan, 2016). Psychologists consider GSE an important mediator between individual behaviors and outcome variables (Tian and Deng, 2011). Previous studies suggest a potential link between GSE and improvements in PR. Enhanced GSE may reduce depression and anxiety in populations (Zhu et al., 2024; Zhang et al., 2024). Individuals with high self-efficacy tend to set more ambitious goals, exert greater effort to achieve them, and possess stronger confidence in their ability to succeed. Compared with individuals with low GSE, those with high GSE hold more optimistic attitudes toward uncertain future events and are more likely to experience positive emotions (Liang, 2019). Research indicates that active participation in physical exercise significantly enhances GSE among university students (Liu, 2020). Related studies suggest that GSE may be associated with exercise duration and intensity (Wang et al., 2015). University students with high GSE are more likely to exert greater effort and persist in various forms of exercise, persevering through difficulties due to their belief in the long-term physical and mental health benefits of exercise (Jin and Dong, 2023; Bandura et al., 1999). Social cognitive theory posits that GSE serves as a crucial mediator between behavioral persistence and psychological adaptation. Therefore, GSE may mediate the relationship between EA and PR. Based on the above analysis, the following hypothesis is proposed:

*Hypothesis H2*: General Eelf-Efficacy exerts a significant positive mediating role in the statistical association between exercise adherence and psychological resilience.

### The mediating role of generalized anxiety symptoms between exercise adherence and psychological resilience

Generalized Anxiety Disorder (GAD) is one of the most prevalent anxiety disorders among patients visiting general hospitals in China, with high prevalence rates observed in general hospitals both domestically and internationally. It is associated with an increased risk of somatic symptoms and sleep disturbances (Kroenke et al., 2007; Xinqiao, 2020). In this study, we focus on the severity of generalized anxiety symptoms (GAS) rather than clinical diagnosis. The term “GAD” is used to refer to the level of anxiety symptoms as measured by the GAD-7 scale. Although only persistent and severe anxiety qualifies for a diagnosis of GAD, subclinical anxiety symptoms that do not meet the diagnostic threshold can also cause multifaceted harm to an individual’s development. Anxiety, as a common psychological issue among university students, consistently shows a high detection rate (Li et al., 2011). In recent years, the anxiety levels among university students in China have shown a rising trend (Chen et al., 2022; Li et al., 2023). The causes of anxiety among university students are diverse, potentially involving academic pressures, family issues, economic factors, and other aspects (Aktekin et al., 2001). Previous research indicates that university students at higher risk of anxiety often exhibit lower levels of mental health (Peng et al., 2024). Exercise, as an active lifestyle choice, can provide individuals with positive emotional experiences, alleviate negative emotions such as anxiety and depression, and thereby enhance mental health (Sun et al., 2019; Xiang et al., 2024). Sustained and regular physical exercise can not only improve an individual’s physical and mental health, enhance physical fitness, and boost immunity, but also effectively mitigate anxiety and depression through interactions between physiological and psychological mechanisms, thereby enhancing PR (Sun et al., 2024; Guo, 2024). According to the Attention Diversion Theory, when individuals redirect their attentional resources from persistent internal worries and negative thoughts toward sustained, regular physical exercise, they can effectively disrupt anxiety, thereby reducing the level of GAS. Therefore, GAS may play a mediating role between EA and PR. Based on the above analysis, the following hypothesis is proposed:

*Hypothesis H3*: Generalized anxiety symptoms exert a significant negative mediating role in the statistical association between exercise adherence and psychological resilience.

### The serial mediating role of general self-efficacy and generalized anxiety symptoms

General self-efficacy theory, proposed by the American psychologist Bandura, emphasizes an individual’s belief in their ability to accomplish specific tasks, which is a key determinant of behavioral choices, effort, and persistence (Bandura et al., 1999). GSE, as an individual’s belief in their own capabilities, can accumulate during exercise and help alleviate anxiety. Meanwhile, the mechanism of attention diversion helps individuals disengage from anxious thoughts. GSE can be progressively built through exercise, thereby mitigating GAD and ultimately enhancing an individual’s PR (Spitzer et al., 2006; Connor and Davidson, 2003). Attention diversion theory posits that when individuals engage in attention-demanding activities, they can shift their focus away from negative emotions or thoughts, thereby temporarily alleviating emotional distress (Nolen-Hoeksema and Lyubomirsky, 2008). EA, as a form of regular physical activity that requires focused attention, can effectively occupy cognitive resources, interrupt anxiety-related thoughts, and help individuals detach from a state of persistent emotional arousal. Sustained practice of attention diversion may reduce an individual’s focus on psychologically threatening information, thereby lowering the level of GAS. This study posits that EA may be positively associated with university students’ GSE by accumulating efficacy beliefs emphasized in general self-efficacy theory. According to general self-efficacy theory, sustained exercise adherence provides individuals with repeated mastery experiences, which enhance their belief in one’s capability to execute actions required to manage prospective situations. Within the attention-diversion framework, this enhanced GSE belief may positively relate to individuals’ proactive attentional diversion and reduce the processing of anxiety-provoking information, thereby reducing GAS among university students. Ultimately, this process improves university students’ PR through a serial mediation pathway (Bandura et al., 1999; Masten, 2001). Based on general self-efficacy theory and attentional diversion theory, this study proposes that EA among university students may be positively associated with PR by first increasing GSE, which, in turn, reduces GAS. GSE and GAS may play a serial mediating role between EA and PR, and accordingly, a serial mediation model was constructed. This paper aims to comprehensively explore how EA influences university students’ PR, offering theoretical support and practical recommendations to enhance their physical and mental health. Based on the above analysis, the following hypothesis is proposed:

*Hypothesis H4*: General Self-Efficacy and generalized anxiety symptoms exert a significant serial mediating role in the statistical association between exercise adherence and psychological resilience.

**Figure 1:**
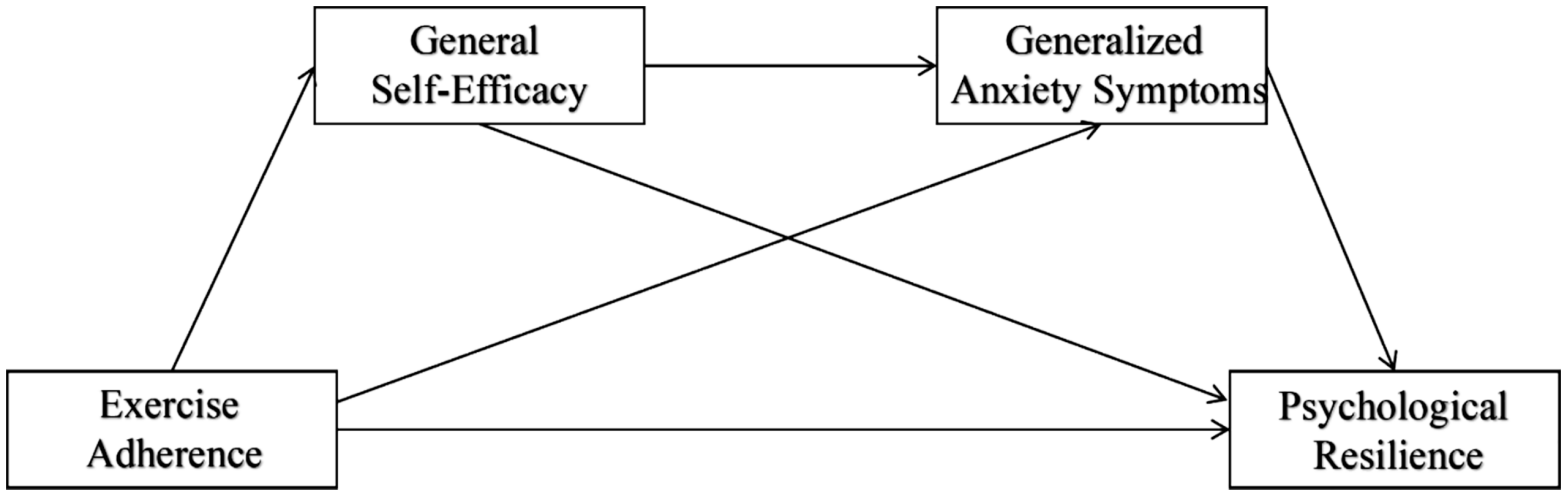
Diagram of the hypothetical model.

Integrating the above analysis and hypotheses, this study takes enrolled university students in Chinese higher education institutions as the survey subjects, with EA as the independent variable and PR as the dependent variable. A serial mediation model (as shown in Figure 1) is constructed based on general self-efficacy theory and attention-diversion theory to examine the impact of EA on the PR of university students and its underlying mechanisms, aiming to provide theoretical guidance and empirical support for promoting the physical and mental health development of university students.

## Methods

### Participants

This study conducted a sampling survey using epidemiological survey methods, employing a stratified, cluster-based, and multi-stage sampling approach to select participants. The survey targeted undergraduate and junior college students enrolled in regular institutions of higher education in China, excluding postgraduate students (master’s and doctoral). The specific sampling procedures were as follows:

#### Determining sampling sites

To ensure the representativeness of the monitored population, each province (autonomous region, municipality) was allocated an average of three sampling sites, with an equal number of samples drawn from different cities. The detailed procedure was as follows: Prefecture-level cities within each province or autonomous region served as potential sampling sites. Among these, the provincial capital city was designated as a “Type I” sampling site. The selection principles for the other two sites were: considering the geographical distribution within the province/autonomous region, one city with an average level of socioeconomic development was selected as a “Type II” sampling site, and one city with a relatively lower level of socioeconomic development was selected as a “Type III” sampling site. For municipalities directly under the central government, the sample selection did not adhere to the aforementioned principles but was primarily based on random cluster sampling, while still maintaining the principle of having three sampling sites.

#### Determining sampling units

The selection of sampling units was primarily based on four considerations: first, the institution of higher education must be a formally established one registered with the Ministry of Education, including vocational colleges; second, the unit must be able to meet the sampling requirements (e.g., age, number of students, grade distribution); third, the unit must have a designated person responsible for questionnaire distribution and be willing to participate in long-term monitoring; fourth, students at the university of the selected sampling unit must have completed their return to campus for the autumn semester.

#### Grouping and sample size

The population was first stratified by gender (biological sex: male, female) into two groups. Each gender group was then further stratified by academic year into four subgroups. Each of these subgroups (e.g., first-year male students) was subsequently categorized into four groups based on sleep quality (very good, fairly good, average, poor). Data for this study were collected online via questionnaires, with researchers and supervising instructors present during the sessions. At the beginning of the study, researchers obtained informed consent from participants. These forms clearly outlined the research purpose, methods, potential risks, and participants’ rights, ensuring that participation provided fully informed consent. All participants were informed that the questionnaire would take approximately 12 min to complete and that they could withdraw from the study at any time without any negative consequences. A pilot survey was conducted before the formal questionnaire was administered, and the questionnaire design was optimized based on the feedback. Participants’ responses were collected anonymously, and all collected data were kept confidential to mitigate potential self-reporting bias.

It should be noted that: first, data collection for this study was conducted online via questionnaires, with researchers and supervising instructors present; second, university students participated voluntarily and anonymously in the survey using an electronic questionnaire format, where students gathered and completed the questionnaire by scanning a QR code; third, this study was approved by the Ethics Committee of Nantong University. Fourth, the criteria for excluding invalid questionnaires were: (1) a missing response rate of 10% or higher across all items; (2) questionnaires where the full name of the university was unidentifiable; (3) contradictory responses between positively and negatively worded items within scales containing reverse-scored items; (4) patterned or identical responses across one or more scales; (5) given the average completion time of approximately 12 min, questionnaires with completion times falling within the (0, 0.5%) or (99.5, 100%) percentiles were excluded. After excluding invalid questionnaires, a total of 24,979 valid questionnaires were retained. The sample was derived from 30 provinces, autonomous regions, and municipalities directly under the central government in China; the final sample can adequately represent the target population of university students across China. Regarding the relatively low proportion of seniors in the sample, this can be attributed to the fact that senior students face substantial pressures from postgraduate entrance examinations and job-seeking activities, which reduced their willingness to participate in the survey. A considerable number of senior students were engaged in off-campus internships during the data collection period, which limited their availability to complete the questionnaire. The sample distribution is presented in Table 1.

**Table 1:** Sample characteristics table.

Variable	*n*	%
Gender		
Male	9,796	39.2
Female	15,183	60.8
Sleep quality		
Very good	6,279	25.1
Fairly good	12,235	49.0
Average	5,376	21.5
Poor	1,089	4.4
Grade		
Freshman	14,599	58.4
Sophomore	8,108	32.5
Junior	1,640	6.6
Senior	632	2.5
Total	24,979	100.0

### Measurement

#### Exercise adherence

In this study, university students’ EA was measured using the Exercise Adherence Scale developed by Gu in 2016 (Gu, 2016). The scale comprises three subdimensions: “Exercise Commitment” (6 items), “Exercise Initiative” (4 items), and “Exercise Persistence” (4 items), for a total of 14 items. This scale uses a 5-point Likert scoring system, where higher item scores indicate greater EA among respondents. The scale demonstrates a Cronbach’s alpha coefficient of 0.947 for internal consistency, χ2/df = 2.896, CFI = 0.945, GFI = 0.901, and RESEA = 0.069, indicating good reliability and validity. It effectively reflects exercise participation and can serve as a general tool for investigating EA. The Exercise Adherence Scale has been previously validated in Chinese university student populations and demonstrates adequate psychometric properties (Gu, 2016).

#### Psychological resilience

In this study, the PR of university students was measured using the Adolescent Psychological Resilience Scale developed by Hu and Gan (2008). This scale uses a 5-point Likert scoring system, with a total score ranging from 27 to 135. The mean score across all items was calculated, with higher scores indicating greater individual PR. According to research, the scale has a Cronbach’s *α* of 0.80, χ^2^/df = 2.509, CFI = 0.920, GFI = 0.860, RESEA = 0.070, indicating high internal consistency, and can be used as a general tool for studying PR (Smith et al., 2008).

#### General self-efficacy

In this study, GSE was measured using the General Self-Efficacy Scale, developed by Ralf Schwarzer’s team in 1981 (Schwarzer et al., 1995). This unidimensional scale employs a 4-point Likert scoring system. The final score is obtained by dividing the sum of the 10 items by ten. Higher total scores indicate a higher level of GSE. The scale’s established cut-off score is 2.5. Scores below 2.5 indicate low GSE in the participant. An analysis of the scale’s reliability shows a Cronbach’s α of 0.87, test–retest reliability is *r* = 0.83, and split-half reliability is *r* = 0.82, indicating high internal consistency. Therefore, it can serve as a general tool for investigating GSE (Wang et al., 2001).

#### Generalized anxiety disorder scale

In this study, GAD was assessed using the Generalized Anxiety Disorder Scale (GAD-7), which was developed by Spitzer et al. (Spitzer et al., 2006), with a total score ranging from 0 to 21. Higher scores indicate a higher severity of anxiety symptoms in the participants. The GAD-7 measures the severity of anxiety symptoms rather than providing a clinical diagnosis of Generalized Anxiety Disorder. The GAD-7 demonstrates good reliability, with a Cronbach’s α of 0.905, CFI = 0.990, RESEA = 0.060. This scale has shown satisfactory reliability and validity across various studies, establishing it as a valid instrument for assessing anxiety levels in research contexts.

#### Covariates

This study included gender, academic year, and sleep quality as covariates in the analysis. These three variables are closely associated with the physical and mental health of university students. Relevant research indicates that, in terms of gender, female university students are more prone to experiencing depression and anxiety compared to their male counterparts, which may consequently lead to a decline in their mental health status (Twenge and Campbell, 2018). Regarding academic year, students in their lower years, having just entered university, undergo significant psychological changes and exhibit greater emotional volatility, which may contribute to poorer mental health outcomes (Woof et al., 2021). Studies have shown that sleep quality is strongly linked to both EA and psychological well-being among university students (Zhang et al., 2021; Xie et al., 2024). By controlling for these three covariates, the study can more precisely examine the association between EA and PR among university students.

### Statistical methods

In this study, data were primarily processed using SPSS 27.0, Origin, and Excel software. The data are cross-sectional, collected at a single time point, with predictor, mediator, and outcome variables assessed concurrently in November 2024, thereby ensuring temporal consistency. The entire procedure can be categorized into the following steps: (1) Data obtained via “Questionnaire Star” were pre-processed using Excel, including retesting or deleting missing or problematic data. (2) Chi-square tests were employed to analyze differences in EA among students based on gender, academic year, and sleep quality. (3) One-way analysis of variance was used to examine differences in EA, GSE, GAS, and PR among university students across gender, academic year, and sleep quality groups. The η^2^ values range from 0 to 1; according to Cohen’s criteria, values of 0.01, 0.06, and 0.14 represent small, medium, and large effect sizes, respectively (Cohen, 1988). Pearson correlation analysis was conducted to test the relationships among university students’ EA, PR, GSE, and GAS. (4) Mediation effect analysis was performed using regression analysis for exploratory purposes, given the cross-sectional data. A specific mediation analysis was conducted using the Process macro. A 95% confidence interval was set, and the Bootstrap method with 5,000 resamples was used to test mediation effects and explore the underlying mechanisms among the variables.

## Results

### Common method bias

Common method bias was examined using Harman’s single-factor test to detect its potential presence. The results indicated that nine principal components had eigenvalues greater than 1 and were extracted. The largest factor explained 29.834% of the variance, which falls below the commonly used threshold of 40%, confirming the reliability of the data quality. Therefore, this study did not demonstrate significant common method bias.

## Descriptive analysis

As shown in Table 2, the mean EA score for university students was 50.504 ± 9.230. The score for male students was significantly higher than that for female students, and fourth-year students’ scores were notably higher than those of other academic years. For PR, the mean score among university students was 96.062 ± 14.654. At the sleep quality level, the effect size was η^2^ = 0.095, which according to Cohen’s criteria represents a medium effect, which is considerably larger than the effect sizes observed at the gender and academic year levels. The mean GSE score for university students was 18.080 ± 5.453. At the sleep quality level, the effect size was η^2^ = 0.053, which according to Cohen’s criteria represents a small effect, yet it was notably higher than the effect sizes at the gender and academic year levels. The mean GAS score for university students was 10.929 ± 4.273. First-year students scored lower than students in other academic years. At the sleep quality level, the effect size was η^2^ = 0.080, which according to Cohen’s criteria represents a medium effect, which is greater than the effect sizes at the gender and academic year levels. Given the large sample size, statistical significance was near-certain, emphasis should be placed on the effect sizes reported above.

**Table 2:** Descriptive statistics of exercise adherence, general self-efficacy, generalized anxiety symptoms, and psychological resilience among university students with different demographic characteristics.

Variable			Exercise adherence	Psychological resilience	General self-efficacy	Generalized anxiety symptoms
Gender	Male	M	52.886	96.113	18.540	11.021
		sd	9.913	15.558	5.881	4.623
	Female	M	48.967	96.029	17.790	10.869
		sd	8.410	14.039	5.137	4.0300
	η^2^		0.043	<0.001	0.005	<0.001
	F		1121.873	0.195	115.454	7.516
	P		<0.001	0.659	<0.001	0.006
Sleep Quality	Very good	M	53.078	103.107	20.200	9.419
		sd	10.150	16.044	6.075	3.849
	Fairly Good	M	49.929	95.158	17.600	10.853
		sd	8.414	13.214	4.847	3.948
	Average	M	49.186	91.690	17.040	12.120
		Sd	8.955	13.154	5.190	4.401
	Poor	M	48.621	87.176	16.500	14.603
		Sd	10.723	12.533	6.021	5.391
	η^2^		0.028	0.095	0.053	0.080
	F		236.738	875.537	468.830	728.765
	P		<0.001	<0.001	<0.001	<0.001
Grade	Freshman	M	50.624	96.741	17.870	10.659
		sd	8.848	14.522	5.306	4.014
	Sophomore	M	49.995	94.594	18.200	11.300
		sd	9.528	14.440	5.563	4.540
	Junior	M	51.360	96.409	18.990	11.290
		sd	10.412	15.799	5.742	4.721
	Senior	M	52.038	98.300	19.180	11.459
		sd	10.255	15.802	6.182	4.824
	η^2^		0.002	0.005	0.004	0.006
	F		19.611	43.023	32.216	47.220
	P		<0.001	<0.001	<0.001	<0.001
Total		M	50.504	96.062	18.080	10.929
		sd	9.230	14.654	5.453	4.273

### Correlation analyses

**Figure 2:**
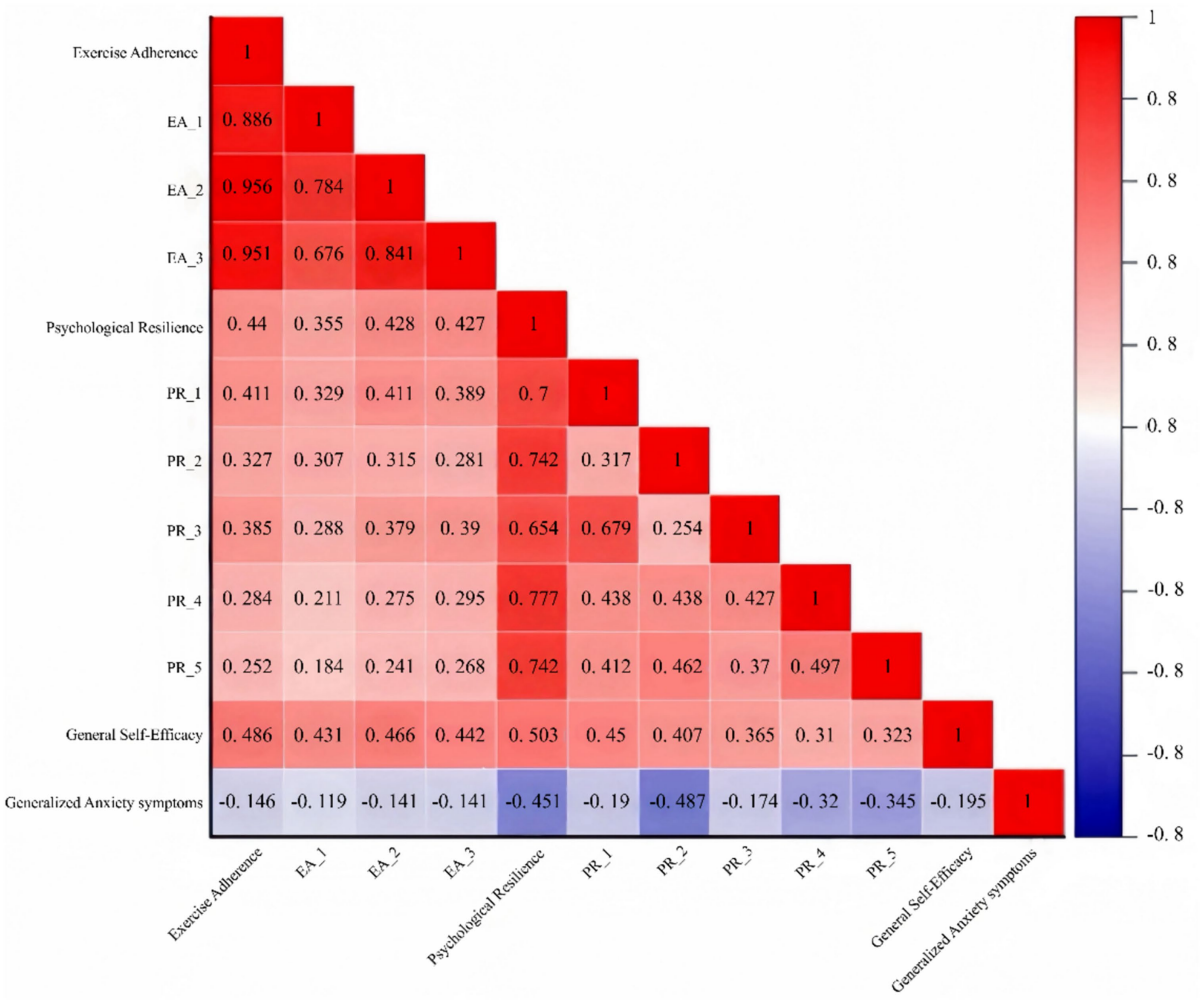
Correlation analyses.

In this study, correlation analyses were primarily conducted using Origin and SPSS 27.0. As shown in Figure 2, there were significant positive correlations between EA and PR, as well as their sub-dimensions, with correlation coefficients ranging from 0.252 to 0.440. A significant positive correlation was found between EA and GSE, with a correlation coefficient of *r* = 0.486 (*p* < 0.001). A significant negative correlation was observed between EA and GAD, with a correlation coefficient of *r* = −0.146 (*p* < 0.001). GSE also showed a significant positive correlation with PR and its sub-dimensions, with correlation coefficients ranging from 0.310 to 0.503. There was a significant negative correlation between GSE and GAS, with a correlation coefficient of *r* = −0.195 (*p* < 0.001). GAS showed significant negative correlations with PR and its sub-dimensions, with correlation coefficients ranging from −0.487 to −0.190.

### Regression analysis

Using SPSS 27.0 and applying Model 6 in the PROCESS macro, further analysis was conducted via the Bootstrap method to test the serial mediation effects. Controlling for gender, academic year, and sleep quality, a serial mediation analysis was performed to examine the mediating roles of GSE and GAS in the relationship between EA and PR. The results, as shown in Table 3, indicate that after controlling for gender, academic year, and sleep quality, EA is positively associated with PR (*β* = 0.382, *p* < 0.001) and GSE (*β* = 0.278, *p* < 0.001), and negatively associated with GAS (β = −0.026, *p* < 0.001). GSE is positively associated with PR (*β* = 0.823, *p* < 0.001) and negatively associated with GAS (*β* = −0.097, *p* < 0.001). GAS is negatively associated with PR (*β* = −1.114, *p* < 0.001). All regression coefficients reflect statistical associations, not causal effects.

**Table 3:** Overview of regression analysis with adjusted confounding factors.

Regression		Fitting indices			Coefficient		
Outcome variables	Predictive variables	*R*	*R^2^*	*F*	*β*	*SE*	*t*
Psychological Resilience	Exercise adherence	0.662	0.438	3244.748***	0.382	0.009	43.245***
	General self-efficacy				0.823	0.015	55.221***
	Generalized anxiety symptoms				−1.114	0.017	−64.757***
	Gender				1.764	0.146	12.083***
	Grade				−0.364	0.096	12.796***
	Sleep quality				−1.945	0.092	−21.007***
General self-efficacy	Exercise adherence	0.509	0.259	2178.826***	0.278	0.003	19.579***
	Gender				0.281	0.062	4.500***
	Grade				0.467	0.041	11.455***
	Sleep quality				−0.941	0.038	−24.925***
Generalized Anxiety Symptoms	Exercise adherence	0.324	0.105	586.112***	−0.026	0.003	−8.078***
	General self-efficacy				−0.097	0.005	−17.837***
	Gender				−0.259	0.054	−4.830***
	Grade				−0.367	0.035	10.426***
	Sleep quality				1.295	0.033	39.370***

*** indicates that the data are significant.

### Mediation effect analysis

As shown in Table 4, the 95% confidence interval for the mediating effect of GSE on the relationship between EA and PR was [−1.080, −1.150]. The confidence interval does not include zero, indicating that GSE plays a significant mediating role in the relationship between EA and PR among university students. EA is associated with higher PR, and this association is partially explained by increased GSE. The 95% confidence interval for the mediating effect of GAS on the association between EA and PR was [0.021, 0.037]. The confidence interval does not include zero, suggesting that generalized anxiety also mediates the relationship between EA and PR. Furthermore, the 95% confidence interval for the serial mediation effect of GSE and GAS on the relationship between EA and PR was [0.026, 0.034], indicating a significant serial mediation effect. EA is positively associated with GSE, which in turn is negatively associated with GAS, ultimately improving PR.

**Table 4:** Summary of mediation effect analysis.

Effect model	Efficiency value	BootSE	LLCI	ULCI	Proportion of total effect
Total effect	0.670	0.009	0.652	0.687	
Direct effect	0.382	0.009	0.364	0.399	57.01%
Indirect effect	0.288	0.007	0.274	0.302	42.99%
Exercise adherence→general self-efficacy→psychological resilience	0.229	0.006	0.217	0.240	34.18%
Exercise adherence→generalized anxiety symptoms→psychological resilience	0.029	0.004	0.021	0.037	4.3%
Exercise Adherence→general self-efficacy→generalized anxiety symptoms→psychological resilience	0.03	0.002	0.026	0.034	4.5%

**Figure 3:**
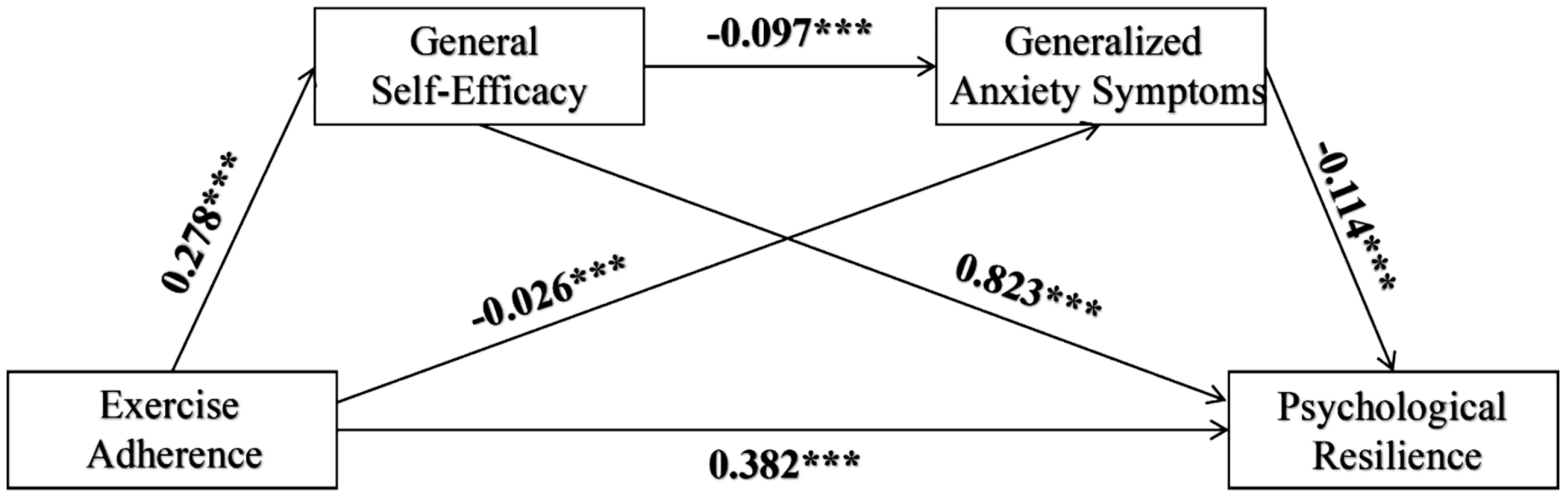
Diagram of mediation effects. *** indicates that the data are significant.

The path coefficient from EA to PR was 0.382 (*p* < 0.001), indicating a significant positive effect. The path coefficient from EA to GSE was 0.278, indicating a significant positive effect of EA on GSE. The path coefficient from EA to GAS was −0.026, indicating a significant negative effect of EA on GAS. The path coefficient from GSE to GAS was −0.097, indicating a significant negative effect of GSE on GAS. The path coefficient from GSE to PR was 0.823, indicating a significant positive effect. The path coefficient from GAS to PR was −0.114, indicating a significant effect of GAS on PR. Note that a higher PR score indicates greater resilience, while a lower score indicates weaker resilience. Higher EA is associated with greater PR, whereas lower EA is linked to weaker PR. Therefore, the mediation effects in this study are illustrated in Figure 3.

## Discussion

Drawing on GSE and attention-diversion theories, this study examined the multidimensional mechanisms underlying the relationship between EA and PR among university students. By introducing GSE and GAS as mediator variables, a serial mediation model linking EA to PR was constructed and tested. The following discussion focuses emphasize practical significance over statistical significance in interpreting the findings. The findings revealed that the total effect of EA on PR was 0.670, comprising a direct effect of 0.382 and an indirect effect of 0.288. This indicates that EA influences PR both directly and indirectly, mediated by GSE and GAS. Given the cross-sectional nature of the data, this model should be interpreted as exploratory rather than confirmatory, all findings should be interpreted as statistical associations rather than causal effects. This study provides a basis for higher education institutions to formulate scientific educational policies and intervention measures, thereby promoting the comprehensive development of university students. The results not only deepen theoretical understanding of the relationship between exercise and mental health but also offer multidimensional practical implications for mental health interventions in higher education settings.

### The positive mediating role of general self-efficacy

The results of this study indicate that GSE plays a positive mediating role in the relationship between EA and PR among university students, confirming hypothesis H2. This finding is consistent with the core proposition of GSE in Bandura’s social cognitive theory, which posits that GSE—an individual’s belief in their own capabilities—serves as a crucial link between environmental challenges and personal adaptation (Bandura, 1999). The results suggest that, in the context of EA, an increase in university students’ GSE is positively associated with their PR, consistent with previous research (Peng et al., 2025). Adherence to physical exercise itself constitutes a process of continuous accumulation. When university students repeatedly achieve exercise goals, they gain direct mastery experiences, which are statistically associated with a strong sense of confidence in their physical capabilities. This confidence may statistically generalize to other areas of life, such as academic pursuits and interpersonal relationships. This generalization effect is associated with students develop higher GSE. Research has shown that the volume of regular physical exercise is significantly positively correlated with university students’ GSE, with students engaging in high-volume exercise demonstrating significantly higher GSE than those engaging in moderate or low volumes (Tong and Ren, 2023). Consistent adherence to exercise is positively related to the formation and enhancement of GSE through repeated mastery experiences. This higher level of GSE is, in turn, positively associated with an individual’s PR in response to common stressors.

### The negative mediating role of generalized anxiety symptoms

This study demonstrates that GAS exerts a negative mediating effect on the relationship between EA and PR among university students, confirming hypothesis H3. The results indicate that, within the context of EA, higher levels of GAS among university students negatively affect their PR, consistent with previous research (Zhang et al., 2022; Xiang et al., 2024; Liu et al., 2024). GAS in China’s current social and educational context, university students face multifaceted pressures, such as postgraduate entrance examinations, employment, and family expectations, that far exceed those of previous generations. Such sustained and intense stressors are likely to trigger GAS. According to attention diversion theory, when exercise is performed as a task that requires cognitive resources, it can effectively divert an individual’s attention away from stressors and negative rumination, which is further associated with higher levels of mood (Ayranci et al., 2025). When an individual experiences anxiety, physical exercise may statistically correspond to a redirection of attention, interrupt negative thought patterns, provide positive mental respite, and create an environment conducive to emotional recovery (Jayakody et al., 2014). Therefore, when EA becomes routine, individuals are more likely to establish a stable, positive psychological environment that gradually and systematically strengthens PR and reduces susceptibility to GAS. From the perspective of general self-efficacy theory, when individuals experience moderate levels of GAS, successfully coping with these anxious experiences can motivate them to mobilize cognitive resources, emotional regulation strategies, and social support networks to address the sources of anxiety. This process gradually shows a positive association with GSE, which is further associated with higher levels of the individual’s ability to maintain mental health and adaptability in the face of stress and adversity—that is, it can exert a certain positive promoting effect on PR. This mechanism is primarily mediated by GSE (Bandura et al., 1999; Wei and Zhang, 2021). Therefore, within the cultural and social context of the study sample, reducing levels of GAS—by facilitating the ongoing development of GSE and providing emotional buffering through attention diversion—lay an important foundation for university students to maintain psychological adaptability when confronting adversity.

### The serial mediating role of general self-efficacy and generalized anxiety symptoms

This study demonstrates that GSE and GAS play a sequential mediating role in the relationship between EA and PR, confirming hypothesis H4. The findings indicate that EA is positively associated with PR by its positive association with GSE, which, in turn, is negatively associated with GAS (Liu, 2020).

According to general self-efficacy theory, an individual’s belief in their own capabilities plays a central role in their behavioral choices and persistence (Bandura et al., 1999). An individual’s behavioral performance depends not only on their actual ability but also on their self-assessment of that ability. This assessment shapes their motivation, level of effort, and persistence when facing challenging situations (Bandura et al., 1999). Individuals with high GSE tend to appraise threatening situations as manageable challenges rather than insurmountable difficulties (Bandura et al., 1999). University students are in late adolescence, a period characterized by the potential for conflict between ego identity and role confusion, a significant source of psychological issues (Itzik and Walsh, 2023). Therefore, when an individual’s GSE is enhanced, they are more likely to view potential stressors as challenges that are not impossible to overcome, thereby reducing their level of GAS in stressful situations. High GSE can positively relate to an individual’s cognitive and behavioral adaptability in the face of adversity, enhancing their goal commitment and persistence in problem-solving. This not only alleviates anxiety but also gradually fosters a more resilient psychological environment. This virtuous cycle ultimately contributes to the beneficial development of PR. General self-efficacy theory also indicates that emotional arousal serves as an important source of information for individuals when making GSE judgments (Bandura et al., 1999). The physiological and psychological benefits of sustained exercise help reduce anxiety-related emotional arousal, with greater benefits accruing from long-term, regular EA.

From the perspective of attention diversion theory, individuals can effectively reduce immediate emotional distress by shifting their attention from negative stimuli or anxious states toward external activities, thereby creating conditions for positive cognitive and behavioral adjustments. Regular EA provides individuals with sustained, cumulative mastery experiences (Nolen-Hoeksema and Lyubomirsky, 2008). Through repeatedly completing exercise activities that present a certain level of challenge, an individual’s confidence in their coping abilities is strengthened, forming positive GSE expectations and behavioral orientations. Attentional diversion theory posits that EA helps individuals develop a stable state of attentional focus (Masten, 2001). When anxiety arises, individuals can proactively direct attentional resources toward the exercise activity itself and its accompanying bodily sensations, effectively interrupting the continuation and spread of anxious thoughts. This process creates a more stable, less distracting psychological environment that is conducive to the formation and reinforcement of GSE. Therefore, EA alleviates immediate emotional distress by diverting attention and, through repeated practice, consolidates GSE and behavioral patterns, thereby countering anxiety and enhancing PR among university students.

## Limitations

This study has several limitations. (1) The cross-sectional design may preclude definitive conclusions regarding causality. In particular, the alternative directional pathway in which psychological resilience and general self-efficacy predict exercise adherence rather than the reverse cannot be ruled out and should be systematically tested in future research using longitudinal or experimental designs. (2) The reliance on self-reported questionnaires introduces the possibility of recall bias and means that common method bias cannot be fully ruled out. More rigorous methods to detect or mitigate such bias were not feasible in this study, largely because the unidimensional nature of the GSE and GAS scales prevented the use of more advanced statistical approaches. Future research should therefore incorporate objective measurement tools (e.g., accelerometers) alongside multidimensional measures or longitudinal designs to enhance both reliability and validity. (3) Social desirability bias may also have influenced participants’ responses, as individuals might overestimate their EA or underestimate negative psychological states to present a more favorable self-image. (4) The study may not have fully accounted for other variables potentially influencing university students’ PR. For instance, factors such as family socioeconomic status, parenting styles, social support networks, and personal character traits, whose impacts were not extensively explored, could affect the precision of the results. (5) Alternative models, including reverse causality (e.g., PR influencing EA) or reciprocal relationships, remain possible and should be tested in future research.

## Conclusion

This study identifies the associations through which EA is significantly linked to PR in university students, operating via both direct and indirect pathways, the proposed serial mediation model assumes a directional relationship from EA to PR via GSE and GAS, but causal mechanisms cannot be determined. The findings hold significant implications for health promotion in higher education institutions. These institutions bear an important responsibility for advancing students’ physical and mental health education. Through comprehensive interventions such as establishing exercise support systems, enhancing GSE cultivation, and providing anxiety counseling, they may be associated with students’ PR and adaptive capacities. The research results provide a theoretical foundation and practical direction for the systematic implementation of PR promotion strategies in universities.

## Data Availability

The raw data supporting the conclusions of this article will be made available by the authors, without undue reservation.
